# Giant Pulmonary Hamartoma in a Child: A Case Report

**DOI:** 10.1111/jpc.70361

**Published:** 2026-03-15

**Authors:** Jurgen Schleef, Marianna Carbi, Valentina Kiren, Marco Rabusin, Sergio Ghirardo, Massimo Maschio, Egidio Barbi, Rossana Bussani, Claudio Granata, Anita Spezzacatene

**Affiliations:** ^1^ Department of Pediatric Surgery Institute for Maternal and Child Health, IRCCS ‘Burlo Garofolo’ Trieste Italy; ^2^ Department of Medical, Surgical and Health Sciences University of Trieste Trieste Italy; ^3^ Department of Pediatrics Institute for Maternal and Child Health, IRCCS ‘Burlo Garofolo’ Trieste Italy; ^4^ Department of Anatomic Pathology Azienda Sanitaria Universitaria Giuliano Isontina Trieste Italy; ^5^ Department of Pediatric Radiology Institute for Maternal and Child Health, IRCCS ‘Burlo Garofolo’ Trieste Italy

**Keywords:** case reports, child, cough, hamartoma, lung

## 1 Introduction

Pulmonary hamartomas are benign, slow‐growing lesions composed of variable proportions of mesenchymal elements. They typically present as well‐circumscribed, solitary nodules and less frequently as masses (> 3 cm). Most pulmonary hamartomas arise in the peripheral lung parenchyma and remain asymptomatic, with only a minority occurring in a central endobronchial location. On chest computed tomography (CT), fat attenuation is observed in approximately 50%–60% of cases, whereas calcifications are variably demonstrated depending on lesion size [1]. They are most often detected incidentally in adults and represent an uncommon consideration in the differential diagnosis of paediatric lung lesions.

We describe an unusual case of a child with a symptomatic, predominantly cystic, air‐filled lung lesion, apparently arising from the left lower lobe as demonstrated on chest imaging. The lesion posed a diagnostic challenge due to overlapping radiological features with other cystic pulmonary lesions.

This manuscript was prepared following the CARE guidelines (https://www.care‐statement.org).

## 2 Case Report

An 11‐year‐old child experienced an acute infectious episode characterised by asthenia, low‐grade fever, rhinitis and cough. Following resolution of the acute illness, the patient continued to have a daily productive cough accompanied by exertional dyspnea and mild chest pain. Initial treatment with oral antibiotics and a corticosteroid nasal spray resulted in only partial improvement, prompting a paediatric pulmonology consultation. On examination, fine crackles were noted at the left lung base. Laboratory tests were within normal limits. Past medical history was unremarkable, and prenatal ultrasound findings were reported as normal.

**FIGURE 1:**
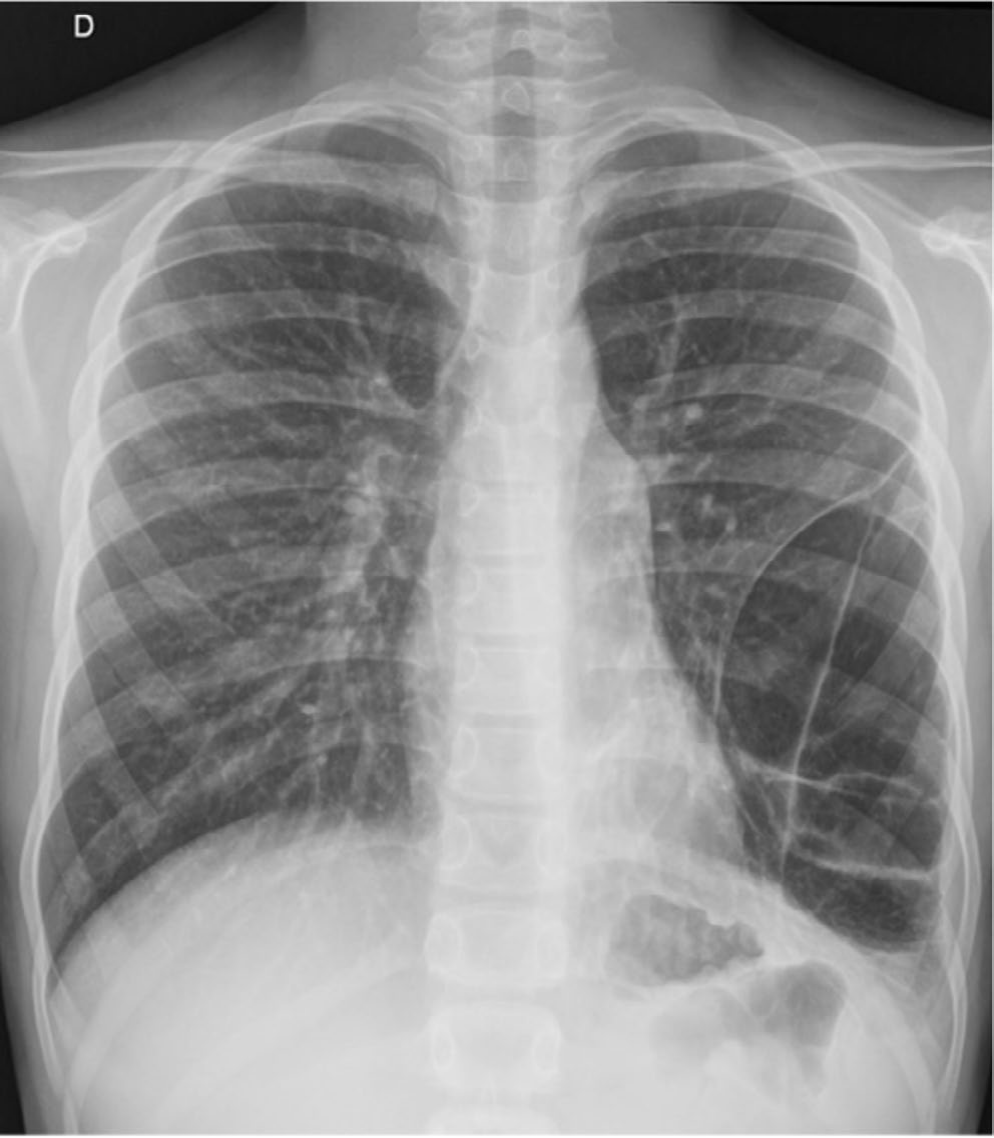
Radiograph (posteroanterior) demonstrates a large hyperlucent area with internal septation in the lower‐middle third of the left hemithorax, consistent with a cystic lesion.

Given the presence of cough for more than 4 weeks, a chest radiograph was obtained, revealing a well‐defined lesion in the middle‐to‐lower third of the left hemithorax. The lesion appeared radiolucent, exhibiting a cystic morphology with internal septations, some of which were thickened. A minimal area of retrocardiac pulmonary parenchymal consolidation was also observed. Overall, the radiographic appearance was suggestive of either a congenital malformation or a neoplastic process (Figure 1).

**FIGURE 2:**
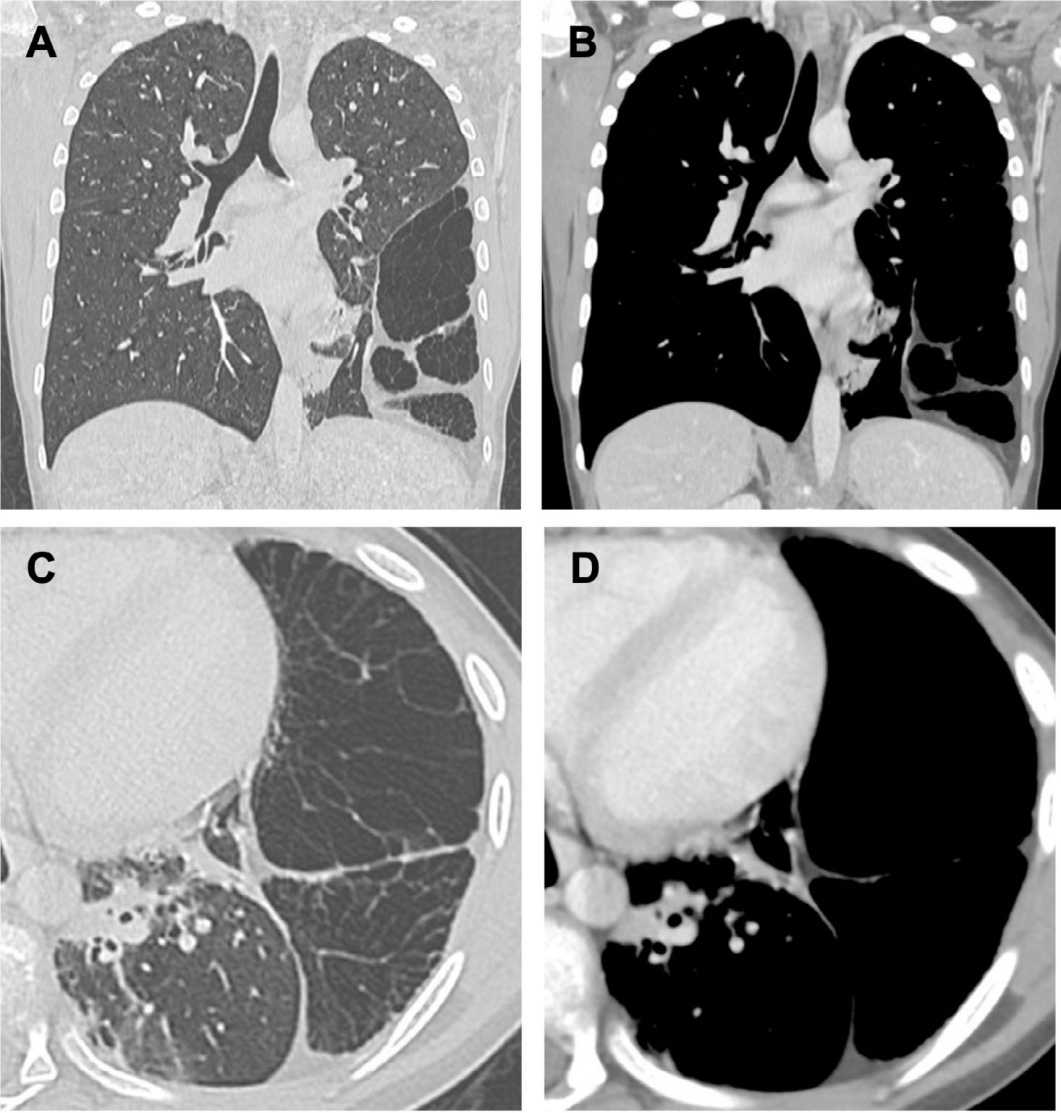
Contrast‐enhanced chest computed tomography: Coronal view (A) lung window showing a pulmonary lesion characterised by air‐filled, cyst‐like spaces, separated by multiple internal septations and (B) soft‐tissue window, better highlighting peripheral adipose component. Axial view (C) lung and (D) soft‐tissue windows.

Contrast‐enhanced CT (CECT) of the chest confirmed the presence of a large peripheral lesion in the middle‐to‐lower third of the left hemithorax, measuring 13 × 5 × 14 cm (w × d × h) and partially compressing the adjacent lingula and lower lobe (Figure 2). The lesion was predominantly composed of air‐filled, cyst‐like spaces separated by multiple internal septations, mostly thin, traversed by scant vascular structures. Along its periphery, the lesion was partially delineated by adipose tissue, from which a few extensions projected inward, interposed amongst the cystic spaces. No calcifications were identified, and there were no pathological lymphadenopathies or other notable findings.

Ongoing symptoms and the uncertain malignant potential of the lesion prompted surgical exploration following multidisciplinary review.

Thoracoscopic exploration revealed a large mass with mixed lardaceous and cystic consistency, apparently connected to the left upper lobe by a thin pedicle forming a partial twist. Aspiration under direct vision yielded only a few millilitres of air, leading to conversion to an open approach and complete excision of the lesion. Haemostasis was secured, and a chest drain was placed. The postoperative course was uneventful.

**FIGURE 3:**
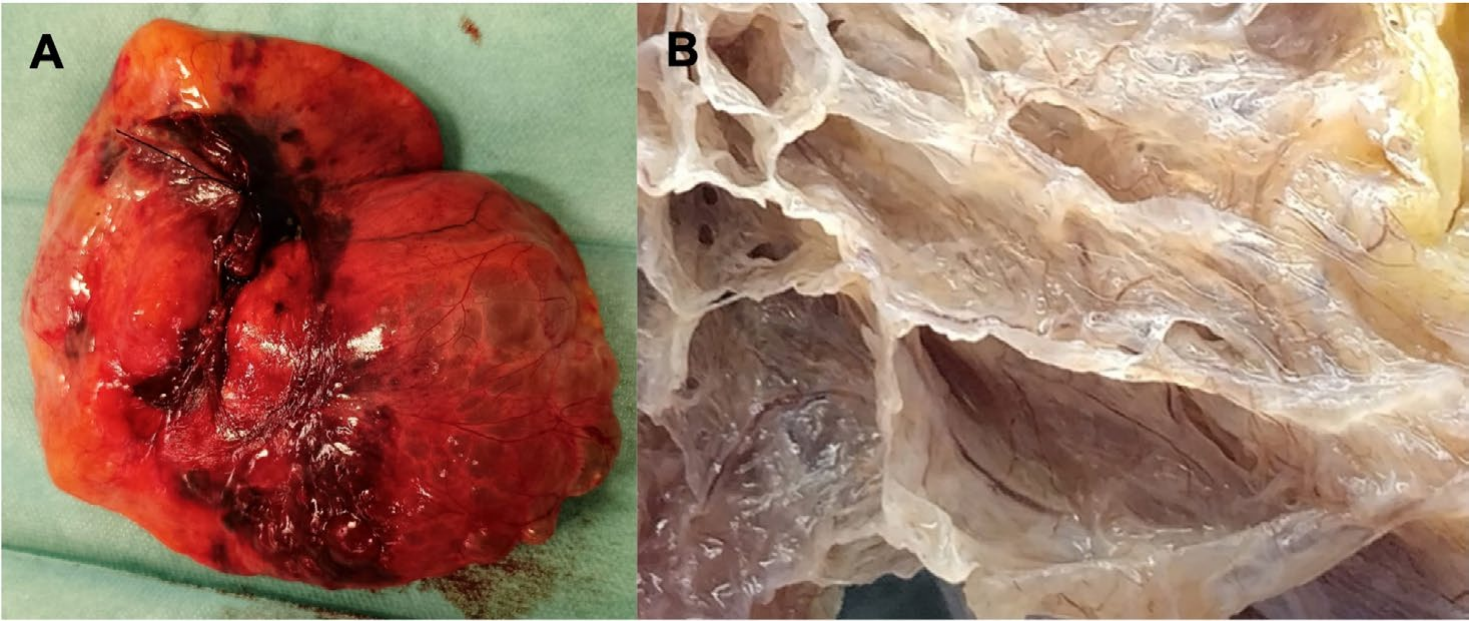
Photographs: Hamartoma after surgical resection (A) and gross examination on cut surface (B) showing multiloculated architecture with thick, greyish fibrous septa.

**FIGURE 4:**
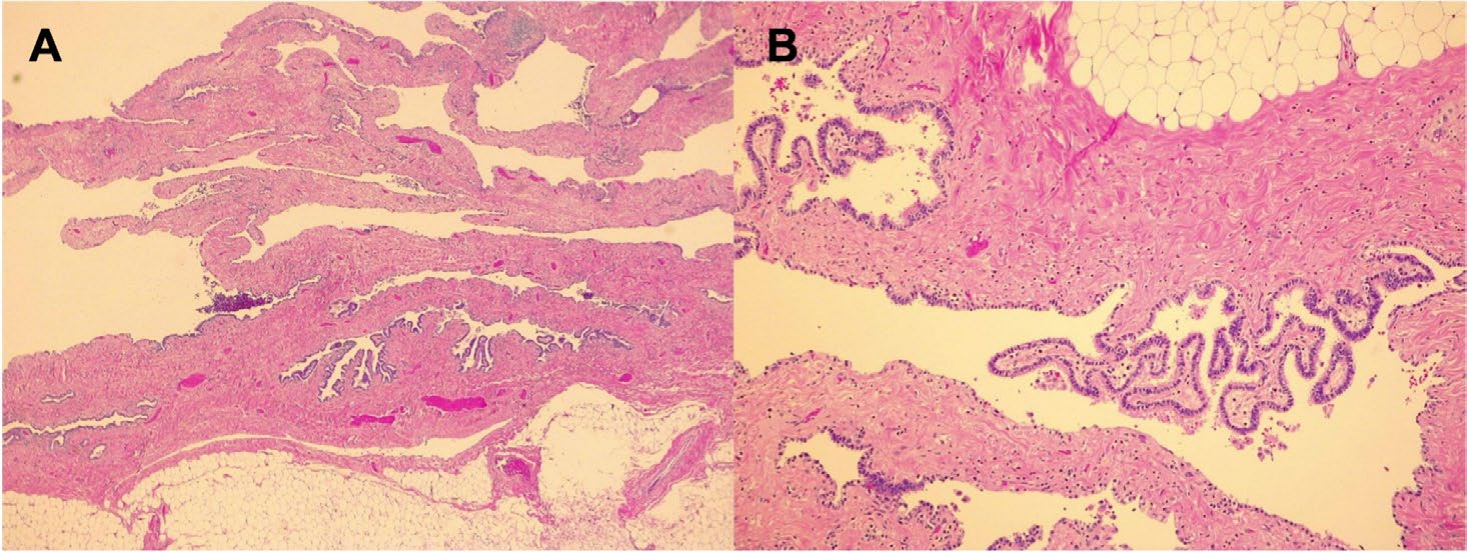
Photomicrographs H&E, ×2.5 (A) and ×10 (B). Fibroadipose digitations that progressively shift into connective‐muscular septae delimited in places by flattened cubic epithelium and in places by cylindrical‐ciliated epithelium without atypia. The septa organise morphologically ‘nonsense’ intermingling, constructing an adenofibrolipomatous pattern and small club‐like papillae embedded in a fibrous stroma.

Gross examination by the pathologist revealed multiple thick, greyish parenchymal septa with a pulmonary‐like architecture, enclosed within a lipomatous capsule (Figure 3), without necrosis or haemorrhage. Microscopically, fibroadipose septa originated from the lipomatous capsule and progressively merged into connective‐muscular structures. These septa were partially lined by flattened cuboidal or ciliated columnar epithelium, occasionally exhibiting small club‐like papillae (Figure 4). No atypia or neoplastic features were observed. Histomorphology was consistent with a giant pulmonary adenofibrolipomatous hamartoma. The lesion was completely excised. Additionally, KRAS mutation analysis was performed and was negative.

## 3 Discussion

Pulmonary hamartomas are common benign lesions in adults but are distinctly rare in children. They are composed of variable amounts of at least two mesenchymal tissues—connective tissue, cartilage, fat and smooth muscle—interspersed with benign respiratory epithelium and occasionally exhibiting cystic changes. Radiological diagnosis of pulmonary hamartoma is possible when characteristic CT features are present, such as a well‐circumscribed nodule, popcorn calcifications and a fat density component [1]. In such cases, they usually remain clinically silent and do not require surgical intervention; however, some peripheral lesions may acquire atypical features, grow considerably in size and become symptomatic.

To our knowledge, only a few cases of large pulmonary cystic hamartomas have been reported in the literature, predominantly in adults [2, 3, 4, 5, 6, 7], with only one case in the paediatric population closely resembling the present one [7]. Of note, these lesions are distinct from pulmonary mesenchymal cystic hamartomas, which contain immature mesenchymal elements. However, large cystic lesions may exhibit a similar appearance whilst containing both mature and immature mesenchymal components [8].

In adults, most cases were asymptomatic or associated with chronic dyspnea. In children, the first giant cystic pulmonary hamartoma described in this age group occurred in a 11‐year‐old and was asymptomatic [4], whereas the other two cases presented with cough and dyspnea: a 10‐year‐old with pneumothorax on imaging [5] and a 5‐year‐old with recurrent respiratory infections [7], similar to the present case.

Most reported cases were of the chondroid type, characterised by cartilaginous components and/or focal calcifications. In contrast, the case described by Louhaichi et al. [7] was unique in being predominantly adenofibromatous and lipomatous, lacking cartilaginous components as in the present case, whilst exhibiting focal calcifications.

In a single case, a pedicle was identified, later shown on histopathology to contain vascular and bronchial structures; however, this lesion was predominantly solid and had only minimal peripheral cystic components [6].

Notably, the mechanism of cyst‐like spaces formation in pulmonary hamartomas is unclear. Proposed explanations include progressive dilatation of epithelial‐lined tubules through a cheque‐valve effect or the expansion of intrinsic cleft‐like spaces into cysts [2]; in our case, no bronchial connection with the cystic spaces was identified.

In general, paediatric cystic pulmonary lesions are diverse and may show overlapping clinical and radiological features, complicating their distinction [9, 10]. The initial differential diagnosis frequently focuses on congenital pulmonary airway malformations (CPAMs), the most common entity, and pleuropulmonary blastoma (PPB), the most concerning. Amongst the congenital pulmonary airway malformations described by Stocker, Type 1 and Type 2 exhibit a cystic morphology. Importantly, Type 2 lesions are associated with bronchial atresia, whereas Type 1 lesions are linked to KRAS mosaicism [11, 12] and may harbour mucinous cell clusters, suggesting a potential risk of malignant transformation, even in the absence of overt atypia [12, 13]. Consequently, molecular testing has emerged as a valuable adjunct in evaluating paediatric cystic lung lesions, supporting both diagnosis and risk stratification [10]. CPAMs are typically detected prenatally or in neonates, although they may rarely present later in childhood with recurrent infections or as incidental findings. In contrast, pleuropulmonary blastoma (PPB) is a rare but aggressive paediatric neoplasm, most frequently associated with DICER1 variants. Its cystic forms include purely cystic Type I and type Ir, characterised by the presence or absence of immature mesenchymal elements, respectively, and mixed Type II lesions. Whilst imaging can provide valuable information, it cannot reliably distinguish CPAMs from PPB in all cases, with PPB typically diagnosed postnatally, most often within the first 7 years of life [14, 15, 16].

In the present case, prenatal imaging was unremarkable, and the lesion remained asymptomatic until later in childhood, which made CPAM less likely; moreover, repeated or chronic infections can alter pulmonary lesion morphology, further complicating accurate characterisation.

For these reasons, pathological examination was essential both to exclude malignant or premalignant transformation and to confirm the completeness of surgical excision, which is indicated in all symptomatic cases, regardless of the presumed nature of the lesion.

In the literature, most reported cases were treated with lobectomy. However, variations in surgical management have been described: in one case, the lesion exhibited fibrous adhesions to the diaphragm and adjacent lung lobes and, after their release, the mass was excised/enucleated without resection of the surrounding parenchyma [5]; in another case, the lesion showed no adhesions to the normal lung parenchyma, apart from the previously described vascular‐bronchial pedicle, which was therefore dissected, allowing complete excision without residual tissue [6].

## 4 Learning Points

Cystic pulmonary lesions may be asymptomatic or manifest with persistent respiratory symptoms in school‐aged children. Clinical factors, including age at presentation, patient history and imaging are essential to guide diagnosis.

Chest radiography is the first line imaging modality to investigate such conditions, but in some cases it lacks specificity and prompts further investigation with contrast‐enhanced CT in order to better delineate lesion location, cystic versus mixed composition, vascular connections and associated anomalies. Nevertheless, imaging alone cannot always provide a definitive diagnosis, making surgical resection and histopathological examination necessary to relieve symptoms, prevent complications and establish a conclusive diagnosis.

This case highlights the importance of considering giant pulmonary hamartoma in the differential diagnosis of paediatric cystic lung lesions, despite its rarity.

## Funding

This work was supported by the Italian Ministry of Health, through the contribution given to the Institute for Maternal and Child Health IRCCS Burlo Garofolo, Trieste—Italy.

## Ethics Statement

As this was an individual case, where the patient's guardian provided consent, no hospital board ethical approval was required. All procedures involving human participants were conducted in accordance with the ethical standards of the IRCSS Burlo Garofolo, and the Declaration of Helsinki (1964), as revised in 2013.

## Consent

Informed consent for the publication of this case has been obtained from the patient's parents or guardian.

## Conflicts of Interest

The authors declare no conflicts of interest.

## Data Availability

Data sharing is not applicable to this article as no datasets were generated or analysed during the current study.
